# Chemical cues and pheromones in the sea lamprey (*Petromyzon marinus*)

**DOI:** 10.1186/s12983-015-0126-9

**Published:** 2015-11-25

**Authors:** Tyler J. Buchinger, Michael J. Siefkes, Barbara S. Zielinski, Cory O. Brant, Weiming Li

**Affiliations:** Department of Fisheries and Wildlife, Michigan State University, East Lansing, MI USA; Great Lakes Fishery Commission, Ann Arbor, MI 48105 USA; Department of Biological Sciences, University of Windsor, Windsor, ON Canada

**Keywords:** Chemical communication, Olfaction, Sensory biology, Integrated pest management

## Abstract

Chemical cues and pheromones guide decisions in organisms throughout the animal kingdom. The neurobiology, function, and evolution of olfaction are particularly well described in insects, and resulting concepts have driven novel approaches to pest control. However, aside from several exceptions, the olfactory biology of vertebrates remains poorly understood. One exception is the sea lamprey (*Petromyzon marinus*), which relies heavily upon olfaction during reproduction. Here, we provide a broad review of the chemical cues and pheromones used by the sea lamprey during reproduction, including overviews of the sea lamprey olfactory system, chemical cues and pheromones, and potential applications to population management. The critical role of olfaction in mediating the sea lamprey life cycle is evident by a well-developed olfactory system. Sea lamprey use chemical cues and pheromones to identify productive spawning habitat, coordinate spawning behaviors, and avoid risk. Manipulation of olfactory biology offers opportunities for management of populations in the Laurentian Great Lakes, where the sea lamprey is a destructive invader. We suggest that the sea lamprey is a broadly useful organism with which to study vertebrate olfaction because of its simple but well-developed olfactory organ, the dominant role of olfaction in guiding behaviors during reproduction, and the direct implications for vertebrate pest management.

## Background

Sensory input from conspecific odors guides decisions for organisms throughout the animal kingdom [1]. Early studies focused on insects, with the first behaviorally active conspecific odorant identified in the silkmoth (bombykol; *Bombyx mori*) [2]. Since then, behaviors mediated by conspecific odors have been described in crustaceans [3], fishes [4], reptiles and amphibians [5], birds [6], and mammals [7], including hypothesized functions associated with reproduction, foraging, conspecific recognition, and predator avoidance [1]. Detection of chemicals can be integrated into the decision making processes of organisms via adaptations in receivers (chemical cues) or both receivers and signalers (pheromones) [1]. While much of our understanding of chemical communication is based upon research on insects, the olfactory biology and ecology of some vertebrates is increasingly understood. In particular, chemical communication in some fishes, including the sea lamprey (*Petromyzon marinus*), is relatively well described [4].

The sea lamprey is a basal vertebrate with a complex life history comprised of distinct larval, juvenile, and adult stages. Larval sea lamprey burrow into stream sediment and filter feed on organic material and microorganisms. Following a larval stage of 3–5 years, sea lamprey undergo a drastic metamorphosis into the juvenile stage, migrate downstream into the Atlantic Ocean or a Laurentian Great Lake, and parasitize on large fish for approximately 1.5 years. Finally, adult sea lamprey migrate into streams during the spring, where a male will construct a nest and later be joined by one or more females, spawn intermittently for a number of days, and die [8]. Olfaction is hypothesized to influence sea lamprey behavior throughout the larval, juvenile, and adult stages [9–11], but only during the terminal adult phase has the role of conspecific odors been evaluated.

**Fig. 1:**
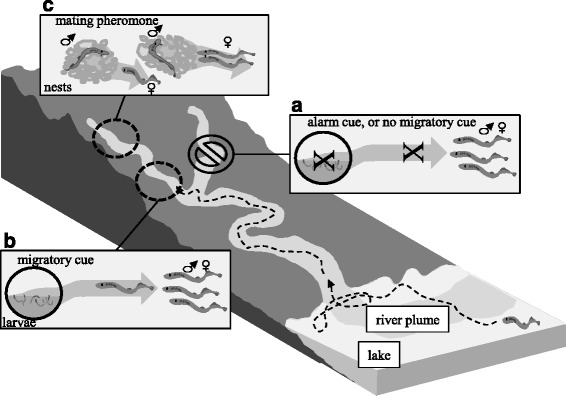
Schematic illustrating the hypothesized functions of migratory cues, alarm cues, and mating pheromones during reproduction in sea lamprey. **a** Fewer migrating sea lamprey enter rivers or tributaries with injured or decaying conspecifics, or lacking larval populations; **b** Migrating sea lamprey enter streams activated with larval odor; **c** upon reaching sexual maturation males release a mating pheromone that draws females to spawning nests, and initiate nest building and spawning behaviors

Adult sea lamprey use conspecific odors to identify suitable spawning habitat, search for mates, and avoid risk (Fig. 1) [11, 12]. Migrating adults select spawning tributaries based upon the odor of previous years’ larvae that reside in the stream. Upon arrival at the spawning grounds, gravid females move upstream and locate spawning nests using the odor of sexually mature males [11, 13]. Alarm substances are hypothesized to guide adults away from areas where larval or adult populations have high mortality [12, 14, 15].

Here, we summarize the current understanding of the chemical cues and pheromones used by the sea lamprey during reproduction. Previous reviews of sea lamprey olfaction focus primarily on applications to fisheries management in the Laurentian Great Lakes [16–20]. Our objective is to develop a broader perspective on sea lamprey olfaction, spanning from odorants up to evolutionary patterns. We provide overviews on the neurobiology of olfaction, the ecology and evolution of chemical cues and pheromones, and potential applications to population management. We suggest that the simple but well-developed olfactory organ, dominant role of olfaction in guiding behaviors during reproduction, and direct implications for vertebrate pest management position the sea lamprey as a useful organism with which to study vertebrate olfaction.

## The olfactory system

### Anatomy of the olfactory apparatus

A critical role of olfaction in mediating the sea lamprey life cycle is evident by a well-developed olfactory system [21]. The large olfactory organ in sea lamprey [22] is comprised of a peripheral olfactory organ containing both a main olfactory epithelium and tubular diverticula known as the accessory olfactory organ [23]. Early in their life cycle, prior to leaving the spawning nest, sea lamprey possess functional olfactory sensory neurons that are stimulated by conspecific odorants [9, 24]. During the metamorphosis from larvae into adults, the peripheral olfactory organ enlarges while changing from an epithelial lined tube to a nasal sac with lamellar folds [25]. The accessory olfactory organ also exhibits the formation of diverticula surrounded by blood vessels and nerve bundles [23, 25].

### Olfactory sensory neurons

Olfactory sensory neurons intercept odor information using dendrites that extend into the mucus of the peripheral olfactory organ. The olfactory sensory neurons are ciliated [24, 26, 27], but exhibit distinct morphotypes similar to ciliated, microvillous, and crypt olfactory sensory neurons documented in teleost fishes [28–30]. Neuron morphotypes differ in the distance the dendrite extends into the olfactory mucus surrounding the olfactory epithelium, and may relay information from different classes of odorants (feeding, risk, reproduction) [31]. Dendrites of sensory neurons express olfactory receptors, which mark the beginning of signal transduction.

### Signal transduction

Olfactory receptors on the olfactory sensory neurons bind odorants and trigger a signal transduction cascade. Receptor proteins of olfactory sensory neurons are members of the seven-transmembrane G-protein coupled receptor superfamily [32]. In the sea lamprey, chemosensory receptor genes include at least 27 olfactory receptor (OR)-type genes, 28 trace amino acid receptors (TAAR)-type and 4 vomeronasal type one (V1R)-type genes [33–35]. Signal transduction following odorant binding is not yet fully described in lamprey. On the main olfactory epithelium, the binding of an odorant by an OR likely triggers a second messenger cascade via the G-protein G_αolf_, which stimulates an increase in cyclic adenosine monophosphate (cAMP), opening the cyclic nucleotide gated ion channel [36–38]. The G-proteins in the olfactory sensory neurons on the accessory olfactory organ, however, have not been identified. The signal transduction cascade leads to depolarization of the neuron and propagation of the signal to the olfactory bulb [36, 37].

### Olfactory bulb

**Fig. 2:**
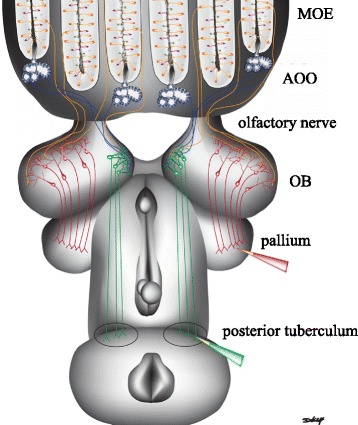
Schematic illustrating the hypothesized circuitry of the sea lamprey olfactory system. MOE = main olfactory epithelium; AOO = accessory olfactory organ; OB = olfactory bulb. Neuronal projections are based upon Ren et al., [27] and Derjean et al., [40]. The medial region of the olfactory bulb receives inputs from the accessory olfactory organ (AOO – blue) as well as sparse inputs from the main olfactory epithelium (MOE – orange). The medial projection neurons (green) project their axons to the posterior tuberculum (PT). The non-medial region of the olfactory bulb receives inputs from the main olfactory epithelium and the non-medial projection neurons (red) project their axons to the pallium. Red and green pipettes indicate location Green et al., [42] injected biocytin to retrogradely label projection neurons in the olfactory bulb (OB). This image is previously published in Green et al., [42]

Spatially distinct regions of the olfactory bulb receive and integrate olfactory signals from the main and accessory olfactory systems (Fig. 2). Olfactory sensory neuron axons projecting from the main olfactory epithelium and the accessory olfactory organ merge into the olfactory nerve. Axons from the accessory olfactory organ project to the medial region of the olfactory bulb, while axons from olfactory sensory neurons in main olfactory epithelium extend to all other regions [27]. After entering the olfactory bulb, olfactory sensory neuron axons pass through the olfactory nerve layer and form synaptic contacts in spherical regions of neuropil known as glomeruli. Glomeruli in all regions, except the medial region, express immunoreactive G_olf_, a G protein thought to be necessary for odorant reception [38]. Within the glomeruli, axon terminals of the olfactory sensory neurons synapse with the dendritic endings of output neurons (projection neurons). Projection neurons in the medial olfactory bulb are spatially isolated from projection neurons in non-medial olfactory bulb regions and have larger cell bodies than non-medial projection neurons [39]. Lastly, projection neurons interact with interneurons and signal higher olfactory processing centers in the brain.

### Projections to the brain and behavioral output

Projections from the medial olfactory bulb to higher olfactory processing centers create a direct link between olfactory input and locomotory output [40]. Odorant and electrical stimulation of the medial region of the olfactory bulb stimulates locomotion [40]. The medial region of the olfactory bulb projects to the posterior tuberculum, which is located in the ventral diencephalon and projects to the mesencephalic locomotor region. The mesencephalic locomotor region initiates locomotion by acting on brainstem pre-motor neurons, the reticulospinal neurons, which directly activate the locomotor networks of the spinal cord [41]. Hence, a direct pathway from a sensory neuron up to the spinal cord likely triggers odor-driven behavioral responses in sea lamprey [40].

In contrast, projections from non-medial regions may be involved in the integration of odor information. Non-medial output neurons project to several forebrain structures, including the lateral pallium. The somata of non-medial projection neurons are below the glomerular neuropil and are smaller than the somata of the medial projection neurons [39]. The receptive fields of the projection neurons in the medial and non-medial output pathways do not overlap [39]. Local field potential recordings from the non-medial olfactory bulb region have shown that the dorsal olfactory bulb territory responds to lamprey sex pheromones and migratory pheromones while lateral olfactory bulb recordings exhibit responses to basic amino acids, and not to pheromones [42]. The hypothesized olfactory-locomotor link created by the accessory olfactory organ may be modulated by the detection and discrimination of specific odorants in the main olfactory organ.

### Olfaction in lamprey compared to other vertebrates

The lamprey olfactory system exhibits many features common among vertebrates, along with several characteristics that are unique. Most organisms, including lamprey, possess similar adaptations for detecting and processing olfactory stimuli [43]. For example, the cellular and molecular mechanisms of olfaction appear to be generally shared among vertebrates, including lamprey; olfactory receptors are G protein-coupled receptors and similar transduction pathways carry olfactory signals [43]. A detailed report of the similarities and differences between the olfactory systems of lamprey and other vertebrates is outside the scope of this review, but several examples of unique features of the lamprey olfactory system should be noted. First, lamprey, along with hagfish, are unique in having a single nostril. Notably, although lamprey have a single nostril, the olfactory organ is comprised of two regions and a paired olfactory nerve. While the functional implications of having a single nostril are unclear, having two nostrils has clear adaptive significance in some fish [44]. Second, the accessory olfactory organ of lamprey appears to be a unique adaptation [35], and offers an interesting comparison to the vomeronasal organ in tetrapods. Taken together, the common and unique features of the sea lamprey olfactory system offers a useful system to answer fundamental questions of vertebrate olfaction.

## Chemical cueing and pheromone communication in sea lamprey

Reproductive behaviors in sea lamprey rely largely upon olfactory input [45, 46]. In contrast to many anadromous fishes (e.g. salmonids), sea lamprey do not exhibit natal homing behaviors [47, 48]. Rather, sea lamprey evaluate the suitability of a stream based on the presence of larval populations [11, 49]. Migratory sea lamprey are acutely tuned to the larval odor (*migratory cue*); putative components are detected at low concentrations [50] and larval odor elicits behavioral responses at the concentrations produced by a single larvae diluted several thousand fold [51]. Once sea lamprey arrive at the spawning grounds, final sexual maturation is partially triggered by conspecific odors [52, 53]. Upon complete sexual maturation, mate search and spawning are guided by the odors of the opposite sex [11]. Although males are attracted to the odor of females [11, 54], the odors released by males (*male mating pheromone*) and subsequent behavioral responses in females are better understood. The male odor appears multi-functional, mediating upstream movement behaviors [55] and proximate nest construction and spawning synchronization behaviors [56]. Finally, throughout the spawning season, sea lamprey are hypothesized to evaluate risk using conspecific and heterospecific semiochemicals (a*larm cue*) [12, 14, 15].

## Migratory cues and mating pheromones

### Identities

**Fig. 3:**
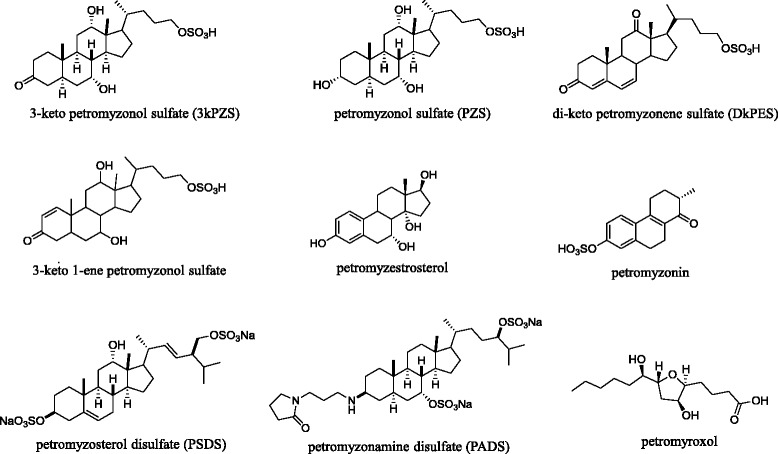
Structures of molecules hypothesized to be behaviorally active pheromones in sea lamprey (*Petromyzon marinus*)

Bile acids and derivatives are implicated as components of the sea lamprey migratory cue and male mating pheromone [19]. The olfactory epithelium of many fishes is sensitive to sex steroids, prostaglandins, amino acids, and bile acids [57]. Sex steroids and prostaglandins are commonly implicated as mating pheromones in teleosts [58]. Amino acids are likely used by anadromous salmonids during natal homing [59, 60], and as a mating pheromone in at least one species (Masu salmon, *Oncorhyncus masou*) [61]. Sea lamprey, however, only show sensitivity to a small number of amino acids and sex steroids [62]. In many species, including sea lamprey, conspecific-released bile acids evoke strong physiological responses in electro-olfactograms (EOG) [50, 63–65], thus implicating behavioral functions. High-performance liquid chromatography (HPLC) and mass spectrometry (MS) combined with EOG screening and behavioral assays have continued to amass support for bile acids and related cholesterol derivatives as components of the male mating pheromone in sea lamprey (Fig. 3) [66].

Research into the sea lamprey migratory cue provides support for the hypothesis that conspecific bile acids [50] guide spawning migrations of anadromous fishes [67]. Larvae excrete lamprey-specific bile acids [68] into the water at rates sufficient to create a detectable concentration in a river (~10 ng/h) [69, 70]. Three bile acids, petromyzonol sulfate (PZS), petromyzonamine disulfate (PADS), and petromyzosterol disulfate (PSDS) are released into the water [70], elicit strong electrophysiological responses from the olfactory epithelium [50, 65], and influence the behavior of migratory lamprey in laboratory mazes [65, 71]. While the mixture of PADS, PSDS, and PZS replicates the proximal preference elicited by larval odor in laboratory tests [65] and may influence search behavior at the junction of the lake and the river [72], the mixture does not replicate larval odor in eliciting upstream movement and stream channel preference in natural stream environments [73], suggesting crucial components of the migratory cue remain unidentified. Several additional components of larval metabolites have been identified and are potent odorants, but have not been evaluated in behavioral assays [74–76].

The first link between bile acids and reproduction was revealed by the discovery that a bile acid functions as a major component of the sea lamprey male mating pheromone [77]. The bile alcohol 3keto petromyzonol sulfate (3kPZS) is released at high rates by males (~0.5 mg/h) [78], detected with acute sensitivity and specificity [64], and elicits an attraction response in sexually mature females both in the laboratory [54, 77] and in the field [54, 55, 77]. While robust behavioral responses in large-scale field tests confirm that 3kPZS is the major component of the male pheromone [55, 79], unknown components appear to be required to match the full suite of nesting and courtship behaviors elicited by the full male odor [56]. A bile acid structurally similar to 3kPZS but lacking in the C24 sulfate, 3 keto allocholic acid (3kACA) was hypothesized to function as an additional component [64, 80, 81], but has now been resolved behaviorally inactive [56]. Notably, sea lamprey detect 3kACA with high sensitivity and specificity [64], and steroidogenesis in males is inhibited by exposure to 3kACA [53]. A 4 oxidized, unsaturated compound similar to 3kPZS elicited attraction in females [82]. Another bile acid 3,12-diketo-4,6-petromyzonene-24-sulfate (DkPES), is a potent male odorant that, when mixed with 3kPZS, increases the number of females that approach the source of 3kPZS [66]. An additional constituent of the male odor, petromyzestrosterol, elicits olfactory responses in EOG recordings but has not yet been tested in behavioral assays [66].

### Sources and release

Sea lamprey possess unique mechanisms of synthesizing and excreting bile acids associated with chemical cues and pheromone. Larval sea lamprey regulate bile acids as do most vertebrates: synthesis in the liver, storage in the gall bladder, and secretion into the intestine via the bile duct. At this stage, putative migratory cue components, including the mating pheromone 3kPZS [83], are slowly released into the water via intestinal contents (~10 ng/larva/h) [69, 70, 83]. A drastic reduction both in expression of genes coding for bile acid biosynthetic enzymes in the liver [83] and in the concentration of bile acids in tissues follows the transformation of larvae into parasitic adults [69, 83]. Migratory adults likewise exhibit a down-regulation of hepatic synthesis of bile acids, but appear to regulate bile acid equilibrium through renal excretion [84]. Upon sexual maturation, males up-regulate expression of genes coding for enzymes involved in bile acid anabolism, yielding an increase in hepatic concentrations of PZS and 3kPZS [77, 78, 85]. The compounds are carried by the cardiovascular system to the gills, where PZS is hypothesized to be oxidized to 3kPZS, and released through glandular cells that develop at the final stages of maturation in males [78, 85]. Additional components of the male pheromone are likely also released by the gills [56]. The cessation of feeding and atrophy of the intestine during reproduction may favor the renal system and gills as alternative mechanisms of bile acid equilibrium.

### Behavioral ecology

The migratory cue informs migrating sea lamprey regarding potential offspring success and reduces the risk of selecting poor stream habitat. Following host detachment, sea lamprey are hypothesized to identify productive offspring habitat using a series of environmental cues [73]. Adult sea lamprey search for river plumes extending into the lake or ocean and display a preference for the general odor of stream water [45, 86]. Migrating adults enter rivers and tributaries that are activated with the odor of larvae, which is directly related to potential for future offspring success [51, 86, 87]. Release of bile acids hypothesized as components of the migratory cue is linked to larval feeding [69, 70]. Although the migratory cue appear to be comprised of a mixture of multiple known and unknown components [65, 73], the functional differences between components is unknown.

The male mating pheromone mediates pre-spawning upstream migration [88] and sexual maturation in males and females [52], and spawning upstream movement [54] and a suite of spawning behaviors in females [56]. The response elicited depends upon the spatial, environmental, and physiological context. Pre-spawning upstream migration of males and females is reduced at low temperatures [89, 90], but maintained in the presence of 3kPZS [88]. Mature male odor facilitates sexual maturation of males and females [52]. Sexually mature females display strong odor-conditioned rheotaxis in response to male odor, primarily in response to 3kPZS [54–56]. Upon reaching the spawning nest, however, nest construction and gamete release in females is largely mediated by the mixture of 3kPZS, DkPES, and unknown compounds [56, 66]. The mechanisms through which pheromone mixtures operate in sea lamprey remain unknown, but specific ratios appear to be important [66].

### Evolution

**Fig. 4:**
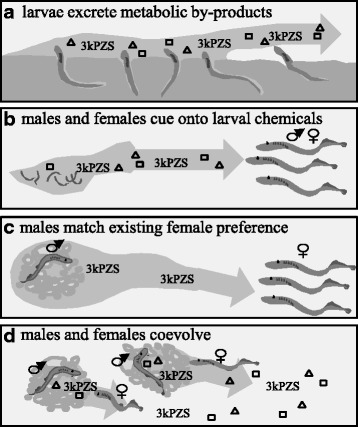
Schematic illustrating the hypothesized evolution of the chemical cueing and pheromone communication systems in sea lamprey. **a** larvae excrete 3-keto petromyzonol sulfate, or 3kPZS, and other chemicals as byproducts of metabolism; **b** migrating adults cue onto 3kPZS and other chemicals to locate habitat conducive to high-offspring survival; **c** males exploit the existing female preference for 3kPZS; **d** male release of 3kPZS continues to become exaggerated as a result of the fitness benefits associated with higher access to mates and female response to 3kPZS transitions from a non-targeted migratory response to a highly proximate spawning response.

The migratory cue appears to be an adaptation of stream-searching adults rather than a specialized signal released by larvae (Fig. 4). Natural selection likely maintains the strong preference for larval odor, where individuals choosing to spawn in streams with clear evidence of historical success realize higher fitness relative to individuals that chose streams at random or undertake high-cost and less effective evaluation of stream habitat via direct assessment [87]. Larvae presumably receive no direct benefit by releasing an attractive odor, thus attraction to larval odor is likely an adaptation of migratory adults [91]. The hypothesis that larval odor represents a cue rather than a signal is supported by the apparent non-specificity of release and response across lampreys [91–96].

The male mating pheromone is likely the result of a more complex evolutionary history. Many fish pheromone systems, including the sea lamprey migratory pheromone [91], appear to represent behavioral adaptations of the receiving fish [91, 97]. Evidence that the silver lamprey (*Ichthyomyzon unicuspis*) uses larval 3kPZS as a migratory cue rather than a male-released mating pheromone suggests female preference for 3kPZS may have originated as an adaptation of receiving fish [96]. The development of glandular cells involved in 3kPZS release [78] and the extremely high rate at which 3kPZS is released [77], however, suggest that male adaptation drove a transition of 3kPZS into a mating signal. Adding further complexity to the mating pheromone is the role of multiple components influencing multiple behaviors [56]. While 3kPZS as a mating pheromone may have evolved through male manipulation of an existing female preference [96], the evolutionary processes driving male release and female preference for the remaining components of the male odor remain unknown.

## Alarm cues

### Identity

Pursuit of the identities of sea lamprey alarm cues is a recent endeavor, and, as such, the chemical structures remain unknown. In fact, despite many years of research on alarm cues in fish, only two alarm odorants have been identified; hypoxanthine-3-N-oxide, an alarm cue in various teleosts [98, 99], and glycosaminoglycan chondroitin, a recently discovered alarm cue in zebrafish (*Danio rerio*) [100]. Although the identity of the sea lamprey alarm cue is uncharacterized, the odor, or part of the odor and is stable past 96 h of aerobic decay [14]. Notably, commercial 2-phenylethylamine (PEA-HCL), a hypothesized predator cue used by rodents [101], elicits an anti-predator response in sea lamprey in the laboratory [15, 102]. Whether the chemically-mediated risk assessment in sea lamprey shows parallels to teleost and other fishes remains unknown.

### Sources and release

Sea lamprey alarm cues originate from conspecific tissues and bodily fluids of predators [14, 15, 102]. Damaged and decayed tissues from larval and adult conspecifics elicit alarm responses [14]. Consistent with much of the literature on fish alarm cues, damaged skin elicits a stronger aversion response compared to whole skin [14]. In contrast to skin-released alarm cues of many fishes [103], the sea lamprey alarm cue appears to be distributed throughout the skin, organ tissue, and muscle [14]. The hypothesized predator cue PEA is released via urine of carnivorous mammals [101] and other unknown predator cues may be released via saliva [102].

### Behavioral ecology

Alarm cues used by sea lamprey could indicate 1) a regional end of the spawning, 2) low offspring survival, or 3) risky spawning habitat [12]. Alarm cues are emitted by both larvae and adults [14], indicating the role of conspecific alarm cues likely spans across the proposed ecological functions. Sea lamprey are semelperous and die following a single reproductive season. Hence, the scent of dead lamprey may indicate the end of the reproductive season in a tributary. Alternatively, alarm cue could indicate low survival of larvae or adults due to poor quality habitat or high predation. Additional functions of alarm cues outside of reproduction are supported by observations of possible alarm responses to damaged conspecific tissue in larval sea lamprey [104]. Larvae also show olfactory sensitivity to odors of non-damaged conspecifics [9]. Whether responses to non-damaged or damaged conspecific odors are ecologically relevant, perhaps influencing settlement behavior, or developmental precursors to the responses during the adult phases remains unknown.

### Evolution

Alarm cues, including sea those used by sea lamprey, are hypothesized to be the result of receiver specializations [103]. Natural selection likely favors an aversion to alarm cues in parallel to the attraction to larval odor, resulting in a multi-faceted mechanism to evaluate spawning habitat and optimize success during the single reproductive event. Notably, sea lamprey also exhibit alarm responses to alarm cue collected from closely related silver lamprey [14], and distantly related white sucker (*Catostomus commersonii*), but not Amazon sailfin catfish (*Pterygoplichthys pardalis*) [15]. Reproductive migrations of sea lamprey, silver lamprey, and white suckers overlap temporally and spatially, hence aversion to alarm cues of heterospecifics is ecologically relevant. However, whether the behaviorally active chemicals are shared across species, or if sea lamprey have evolved to use different compounds released by heterospecific fishes remains unknown.

## Population management

Manipulation of sea lamprey olfactory biology offers opportunities for management of invasive populations in the Laurentian Great Lakes [11]. Based largely upon pheromone control of insects [105], integration of olfactory stimulants into sea lamprey control has been proposed in the forms of trapping, redistribution, disruption, and monitoring [16–19, 106]. However, only trapping has been evaluated in management scale tests [79, 107].

Baiting traps with conspecific odors increases the efficacy of sea lamprey traps [108, 109]. Field experiments in environments lacking background pheromones demonstrate that traps baited with the natural migratory cue and male mating pheromone, and synthesized 3kPZS catch more sea lamprey than unbaited traps [46, 55, 79, 107–110]. However, only 3kPZS has been tested in management-scale experiments, and only in the context of augmenting the existing trapping effort with pheromone as bait [79]. Traps baited with 3kPZS caught more sea lamprey than unbaited traps, and trapping efficiencies averaged about 10 % higher during years when 3kPZS was applied as bait [79]. The modest increase in trapping efficiency is unlikely large enough to justify wide-spread use of 3kPZS as a control measure unless application can be further optimized to improve effectiveness and reduce cost [111]. Notably, the natural, whole odor of males catches a higher proportion of sea lamprey compared to 3kPZS alone [107]. A recent evaluation of the push-pull method using alarm cue to activate one side of a stream and 3kPZS as bait for a trap on the other side of the stream failed to increase the number of sea lamprey caught in traps, although alarm cue did decrease the time taken for individuals to locate a trap [112]. Identification of all components of the male mating pheromone combined with refined trapping methods is needed to further developing odor-baited traps as a control tool.

Methods other than trapping, such as redistribution, disruption, and monitoring [16–19, 106], remain largely unexplored. Field experiments in pristine, odor-controlled environments indicate the migratory cue and male mating pheromone can be used for redistribution [55, 87] and spawning disruption [55]. Redistribution via a combination of conspecific attractants and alarm cues could be especially useful, but has not been evaluated. Quantification of 3kPZS in streams may also offer a cost-effective method to determine the presence and size of sea lamprey populations [113, 114]. Additional alternatives including antagonists [19] and integrating odor manipulation with electrical guidance [115], may too be useful, but have not been explored. Clearly, more research is needed to further develop olfactory cues as tools for sea lamprey control.

## Utility of the sea lamprey model

The sea lamprey presents a simple and unique model for studying olfactory communication in vertebrates. The opportunity for insight into the biology of early vertebrates is matched only by the hagfish. However, the basic biology of sea lamprey is better understood as a result of better accessibility and decades of research associated with pest control programs in the Laurentian Great Lakes. The robust understanding of basic sea lamprey biology combined with the continued elucidation of chemical cues and pheromones, and recent advances, such as the sequencing of the genome [116], allows for novel research avenues.

A simple but well-developed olfactory system makes sea lamprey well-poised for elucidating the path from odorant detection to behavioral output. Sea lamprey detect a limited range of odorants; bile acids, a few amines and sex steroids, and L-arginine [50, 62]. The repertoire of chemosensory receptor genes is correspondingly small, consisting of only three families and an estimated 59 intact genes [34]. Ciliated sensory cells exhibit short, medium, and tall morphotypes that may be precursors to crypt, microvilous, and ciliated sensory cells documented in teleosts [30]. Despite being simple, the sea lamprey olfactory system is well-developed. The distinct accessory olfactory organ with sensory neurons that project to specific regions of the olfactory bulb allows an interesting comparison to the vomeronasal organ of higher vertebrates [27, 35]. The medial olfactory bulb, where the sensory neurons in the accessory organ project axons, forms a direct connection with brain structures that drive locomotion [40]. Strikingly, lamprey have a larger proportion of brain dedicated to processing olfactory information than any other vertebrate examined [22].

Well-adapted mechanisms of habitat and mate assessment using input from multiple olfactory stimuli and environmental cues make sea lamprey a useful organism for studying the evolution and behavioral ecology of multi-model sensory integration and complex signals. During reproduction, sea lamprey make behavioral decisions based upon the water temperature [89, 90], time of day [117], abiotic odor of streams [51], alarm cues [12, 15], multi-component conspecific cues [65, 87] and mating pheromones [17, 55, 56, 66], as well as interactions among variables [88] and the physiological state of the receiver [54]. Furthermore, sea lamprey spawn in lek-like aggregations, where males construct and aggressively defend nests [8], and signal to females with a complex pheromone mixture, setting the stage for studies on the poorly understood role of pheromones in mate choice, the evolution of exaggerated male signals [96], and the function and evolution of multi-component pheromones [56].

Augmenting sea lamprey management with insights from olfactory communication provides a rare example of sensory-integrated control in vertebrates. Manipulation of olfactory systems is a widely used as tools to control pest insect populations [105]. Extension of olfactory integrated control of insects to invasive vertebrates is conceptually sound [16–19, 106, 118], however, after decades of research into fish olfaction, olfactory communication is not integrated into control of any invasive fish. Developing olfactory-integrated management is a challenging and costly endeavor [118], but offers a suite of potentially robust and environmentally benign tools. Olfactory-guided behaviors are not unique to sea lamprey, and insights gained while developing olfactory-integrated control of sea lamprey can be extended to other species of concern throughout the world. Furthermore, the sea lamprey model offers the opportunity to optimize olfactory-integrated control methods without the confounding interactions of other sensory modalities. For example, most organisms, including sea lamprey, incorporate information from several sensory modalities while making reproductive decisions. However, sensory-guided behaviors in sea lamprey are clearly biased towards olfaction [45, 46]. Similar to the insect model used as a conceptual foundation for sea lamprey olfaction research, the sea lamprey model can function as a model for more complex vertebrates. Likewise, the technologies and methods developed for studying sea lamprey olfaction provide a foundation that can be used to expedite future research into olfaction in other organisms that are invasive or in decline in the Laurentian Great Lakes and throughout the world.

## Conclusion

The sea lamprey is a basal vertebrate with an increasingly well-characterized olfactory communication system. We suggest that the olfactory biology of the sea lamprey can be used to inform future research on olfactory systems of other species, as the understanding of the lamprey olfactory biology has been informed by detailed descriptions of olfaction in other organisms. In particular, the simple but well-developed olfactory organ, critical functions of several reproductive chemical cues and pheromones, and potential for population control make studies on sea lamprey olfaction broadly interesting. Current research on sea lamprey olfaction focuses largely on implications for population management [16–20, 106, 118]. However, further research into sea lamprey olfaction, spanning across neurobiology, characterization of chemical cues and pheromones, and ecology and evolution, offers opportunity for a uniquely integrated understanding of chemical communication in a vertebrate.

## Abbreviations

3kPZS: 3keto petromyzonal sulfate
DkPES: Diketo petromyzonene sulfate
PZS: Petromyzonol sulfate
PADS: Petromyzonamine disulfate
PSDS: Petromyzosterol disulfate
2-phenylethylamine: PEA HCL
EOG: Electro-olfactogram
HPLC: High-performance liquid chromatography
MS: Mass spectrometry
cAMP: Cyclic adenosine monophosphate
OR: Odor receptor
V1R: Vomeronasal type 1 receptors
TAAR: Trace amino acid receptors

## References

[1] Wyatt TD (2014). Pheromones and animal behavior: chemical signals and signatures.

[2] Butenandt A, Beckmann R, Stamm D, Hecker ET (1959). Uber den sexual-lockstoff des seidenspinners Bombyx Mori-reindarstellung und konstitution. Z Naturforsch.

[3] Breithaupt T, Thiel M (2011). Chemical communication in crustaceans.

[4] Sorensen PW, Wisenden BD. Fish Pheromones and Related Cues. John Wiley & Sons, Inc., Ames; 2015.

[5] Houck LD (2009). Pheromone communication in amphibians and reptiles. Annu Rev Physiol.

[6] Caro SP, Balthazart J (2010). Pheromones in birds: myth or reality?. J Comp Physiol A.

[7] Tirindelli R, Dibattista M, Pifferi S, Menini A (2009). From pheromones to behavior. Physiol Rev.

[8] Manion PJ, Hanson LH (1980). Spawning behavior and fecundity of lampreys from the upper three Great Lakes. Can J Fish Aquat Sci.

[9] Zielinski BS, Fredricks K, McDonald R, Zaidi AU (2005). Morphological and electrophysiological examination of olfactory sensory neurons during the early developmental prolarval stage of the sea lamprey Petromyzon marinus L. J Neurocytol.

[10] Kleerekoper H, Mogensen J (1963). Role of olfaction in the orientation of Petromyzon marinus. I. Response to a single amine in prey’s body odor. Physiol Zool.

[11] Teeter J (1980). Pheromone communication in sea lampreys (Petromyzon marinus): implications for population management. Can J Fish Aquat Sci.

[12] Wagner CM, Stroud EM, Meckley TD, Kraft C (2011). A deathly odor suggests a new sustainable tool for controlling a costly invasive species. Can J Fish Aquat Sci.

[13] Johnson NS, Buchinger TJ, Li W. Reproductive Ecology of Lampreys. In Docker MF, editor. Lampreys: Biology, Conservation and Control. Springer, Netherlands. 2015; p 265–303.

[14] Bals JD, Wagner CM (2012). Behavioral responses of sea lamprey (Petromyzon marinus) to a putative alarm cue derived from conspecific and heterospecific sources. Behaviour.

[15] Imre I, Di Rocco RT, Belanger CF, Brown GE, Johnson NS (2014). The behavioural response of adult Petromyzon marinus to damage‐released alarm and predator cues. J Fish Biol.

[16] Sorensen PW, Vrieze LA (2003). The chemical ecology and potential application of the sea lamprey migratory pheromone. J Great Lakes Res.

[17] Li W, Siefkes MJ, Scott AP, Teeter JH (2003). Sex pheromone communication in the sea lamprey: implications for integrated management. J Great Lakes Res.

[18] Twohey MB, Sorensen PW, Li W (2003). Possible applications of pheromones in an integrated sea lamprey management program. J Great Lakes Res.

[19] Li W, Twohey M, Jones M, Wagner M (2007). Research to guide use of pheromones to control sea lamprey. J Great Lakes Res.

[20] Siefkes MJ, Steeves TB, Sullivan PW, Twohey MB, Li W. Sea lamprey control: Past, present, and future. In: Taylor WW, Lynch AJ, Leonard NJ, editors. Great Lakes Fisheries Policy and Management. Michigan State University Press, East Lansing. 2013; p 651–704.

[21] Kleerekoper H, van Erkel GA (1960). The olfactory apparatus of Petromyzon marinus L. Can J Zool.

[22] Stoddart DM (1990). The scented ape: the biology and culture of human odour.

[23] Hagelin L, Johnels AG (1955). On the structure and function of the accessory olfactory organ in lampreys. Acta Zool.

[24] VanDenBossche J, Seelye JG, Zielinski BS (1995). The morphology of the olfactory epithelium in larval, juvenile and upstream migrant stages of the sea lamprey, *Petromyzon marinus*. Brain Behav Evol.

[25] VanDenBossche J, Youson JH, Pohlman D, Wong E, Zielinski BS (1997). Metamorphosis of the olfactory organ of the sea lamprey (*Petromyzon marinus* L): Morphological changes and metamorphic analysis. J Morphol.

[26] Thornhill RA (1967). Ultrastructure of olfactory epithelium of lamprey *Lampetra fluviatilis*. J Cell Sci.

[27] Ren X, Chang S, Laframboise AJ, Green W, Dubuc R, Zielinski BS (2009). Projections from the accessory olfactory organ into the medial region of the olfactory bulb in the sea lamprey (*Petromyzon marinus*): A novel vertebrate sensory structure?. J Comp Neurol.

[28] Hansen A, Finger TE (2000). Phyletic distribution of crypt-type olfactory receptor neurons in fishes. Brain Behav Evol.

[29] Hansen A, Zeiske E (1998). The peripheral olfactory organ of the zebrafish, *Danio rerio*: an ultrastructural study. Chem Senses.

[30] Laframboise AJ, Ren X, Chang S, Dubuc R, Zielinski BS (2007). Olfactory sensory neurons in the sea lamprey display polymorphisms. Neurosci Lett.

[31] Lastein S, Hamdani EH, Døving KB. Olfactory discrimination of pheromones. In: Sorensen PW, Wisenden BD, editors. Fish Pheromones and Related Cues. John Wiley & Sons, Inc., Ames; 2015. p159-195.

[32] Buck L, Axel R (1991). A novel multigene family may encode odorant receptors: a molecular basis for odor recognition. Cell.

[33] Grus WE, Zhang J (2009). Origin of the genetic components of the vomeronasal system in the common ancestor of all extant vertebrates. Mol Biol Evol.

[34] Libants S, Carr K, Wu H, Teeter JH, Chung-Davidson YW, Zhang Z (2009). The sea lamprey Petromyzon marinus genome reveals the early origin of several chemosensory receptor families in the vertebrate lineage. BMC Evol Biol.

[35] Chang S, Chung-Davidson YW, Libants SV, Nanlohy KG, Kiupel M, Brown CT (2013). The sea lamprey has a primordial accessory olfactory system. BMC Evol Biol.

[36] Restrepo D, Teeter JH, Schild D (1996). Second messenger signaling in olfactory transduction. J Neurobiol.

[37] Schild D, Restrepo D (1998). Transduction mechanisms in vertebrate olfactory receptor cells. Physiol Rev.

[38] Frontini A, Zaidi AU, Hua H, Wolak TP, Greer CA, Kafitz KW (2003). Glomerular territories in the olfactory bulb from the larval stage of the sea lamprey (*Petromyzon marinus*). J Comp Neurol.

[39] Green WW, Basilious A, Dubuc R, Zielinski BS (2013). The neuroanatomical organization of projection neurons associated with different olfactory bulb pathways in the sea lamprey *(Petromyzon marinus*). PLoS One.

[40] Derjean D, Moussaddy A, Atallah E, St-Pierre M, Auclair F, Chang S (2010). A novel neural substrate for the transformation of olfactory inputs into motor output. PLoS Biol.

[41] Dubuc R, Brocard F, Antri M, Fénelon K, Gariépy JF, Smetana R (2008). Initiation of locomotion in lampreys. Brain Res Rev.

[42] Green WW. The neuroanatomical organization and chemosensory response characteristics of the olfactory bulb in the sea lamprey (Petromyzon marinus). Ph.D. Dissertation. University of Windsor, Windsor, 2013; 226p.

[43] Eisthen HL (2002). Why are olfactory systems of different animals so similar? Brain. Behav Evol.

[44] Velez Z, Hubbard PC, Barata EN, Canário AV (2005). Evidence for functional asymmetry in the olfactory system of the Senegalese sole (Solea senegalensis)*. Physiol Biochem Zool.

[45] Vrieze LA, Bjerselius R, Sorensen PW (2015). Importance of the olfactory sense to migratory sea lampreys Petromyzon marinus seeking riverine spawning habitat. J Fish Biol.

[46] Johnson NS, Luehring MA, Siefkes MJ, Li W (2006). Mating pheromone reception and induced behavior in ovulating female sea lampreys. N Am J Fish Manage.

[47] Bergstedt RA, Seelye JG (1995). Evidence for lack of homing by sea lampreys. T Am Fish Soc.

[48] Waldman J, Grunwald C, Wirgin I (2008). Sea lamprey Petromyzon marinus: an exception to the rule of homing in anadromous fishes. Biol Lett.

[49] Moore HH, Schleen LP (1980). Changes in spawning runs of sea lamprey (Petromyzon marinus) in selected streams of Lake Superior after chemical control. Can J Fish Aquat Sci.

[50] Li WM, Sorensen PW, Gallaher DD (1995). The olfactory system of migratory adult sea lamprey (*Petromyzon marinus*) is specifically and acutely sensitive to unique bile-acids released by conspecific larvae. J Gen Physiol.

[51] Vrieze LA, Sorensen PW (2001). Laboratory assessment of the role of a larval pheromone and natural stream odor in spawning stream localization by migratory sea lamprey (Petromyzon marinus). Can J Fish Aquat Sci.

[52] Chung-Davidson Y-W, Wang H, Siefkes MJ, Bryan MB, Wu H, Johnson NS (2013). Pheromonal bile acid 3-ketopetromyzonol sulfate primes the neuroendocrine system in sea lamprey. BMC Neurosci.

[53] Chung-Davidson YW, Wang H, Bryan MB, Wu H, Johnson NS, Li W (2013). An anti-steroidogenic inhibitory primer pheromone in male sea lamprey (Petromyzon marinus). Gen Comp Endocr.

[54] Siefkes MJ, Winterstein SR, Li W (2005). Evidence that 3-keto petromyzonol sulphate specifically attracts ovulating female sea lamprey Petromyzon marinus. Anim Behav.

[55] Johnson NS, Yun S-S, Thompson HT, Brant CO, Li W (2009). A synthesized pheromone induces upstream movement in female sea lamprey and summons them into traps. Proc Natl Acad Sci U S A.

[56] Johnson NS, Yun S-S, Buchinger TJ, Li W (2012). Multiple functions of a multi-component mating pheromone in sea lamprey Petromyzon marinus. J Fish Biol.

[57] Hara TJ (1994). The diversity of chemical stimulation in fish olfaction and gustation. Rev Fish Biol Fisher.

[58] Stacey N. Hormonally-derived pheromones in teleost fishes. In: Sorensen PW, Wisenden BD, editors. Fish Pheromones and Related Cues. John Wiley & Sons, Inc., Ames; 2015. p33-88.

[59] Shoji T, Yamamoto Y, Nishikawa D, Kurihara K, Ueda H (2003). Amino acids in stream water are essential for salmon homing migration. Fish Physiol Biochem.

[60] Shoji T, Ueda H, Ohgami T, Sakamoto T, Katsuragi Y, Yamauchi K (2000). Amino acids dissolved in stream water as possible home stream odorants for masu salmon. Chem Senses.

[61] Yambe H, Kitamura S, Kamio M, Yamada M, Matsunaga S, Fusetani N (2006). L-Kynurenine, an amino acid identified as a sex pheromone in the urine of ovulated female masu salmon. Proc Natl Acad Sci U S A.

[62] Li W (1994). Olfactory biology of adult sea lamprey (Petromyzon marinus).

[63] Buchinger TJ, Li W, Johnson NS (2014). Bile salts as semiochemicals in fish. Chem Senses.

[64] Siefkes MJ, Li W (2004). Electrophysiological evidence for detection and discrimination of pheromonal bile acids by the olfactory epithelium of female sea lampreys (Petromyzon marinus). J Comp Physiol A.

[65] Sorensen PW, Fine JM, Dvornikovs V, Jeffrey CS, Shao F, Wang J (2005). Mixture of new sulfated steroids functions as a migratory pheromone in the sea lamprey. Nat Chem Biol.

[66] Li K, Brant CO, Siefkes MJ, Kruckman HG, Li W (2013). Characterization of a novel bile alcohol sulfate released by sexually mature male Sea Lamprey (*Petromyzon marinus*). PLoS ONE.

[67] Døving KB, Selset R, Thommesen G (1980). Olfactory sensitivity to bile acids in salmonid fishes. Acta Physiol Scand.

[68] Haslewood GAD, Tökés L (1969). Comparative studies of bile salts. Bile salts of the lamprey Petromyzon marinus L. Biochem J.

[69] Polkinghorne CN, Olson JM, Gallaher DG, Sorensen PW (2001). Larval sea lamprey release two unique bile acids** to the water at a rate sufficient to produce detectable riverine pheromone plumes. Fish Physiol Biochem.

[70] Fine JM, Sorensen PW (2010). Production and fate of the sea lamprey migratory pheromone. Fish Physiol Biochem.

[71] Bjerselius R, Li W, Teeter JH, Seelye JG, Johnsen PB, Maniak PJ (2000). Direct behavioral evidence that unique bile acids released by larval sea lamprey (Petromyzon marinus) function as a migratory pheromone. Can J Fish Aquat Sci.

[72] Meckley TD, Wagner CM, Gurarie E (2014). Coastal movements of migrating sea lamprey (Petromyzon marinus) in response to a partial pheromone added to river water: implications for management of invasive populations. Can J Fish Aquat Sci.

[73] Meckley TD, Wagner CM, Luehring MA (2012). Field evaluation of larval odor and mixtures of synthetic pheromone components for attracting migrating sea lampreys in rivers. J Chem Ecol.

[74] Li K, Siefkes MJ, Brant CO, Li W (2012). Isolation and identification of petromyzestrosterol, a polyhydroxysteroid from sexually mature male sea lamprey (Petromyzon marinus L.). Steroids.

[75] Li K, Brant CO, Huertas M, Hur SK, Li W (2013). Petromyzonin, a hexahydrophenanthrene sulfate isolated from the larval sea lamprey (Petromyzon marinus L.). Org Lett.

[76] Li K, Huertas M, Brant C, Chung-Davidson YW, Bussy U, Hoye TR (2014). (+)-and (−)-Petromyroxols: Antipodal tetrahydrofurandiols from larval sea lamprey (Petromyzon marinus L.) that elicit enantioselective olfactory responses. Org Lett.

[77] Li W, Scott AP, Siefkes MJ, Yan H, Liu Q, Yun S-S (2002). Bile acid secreted by male sea lamprey that acts as a sex pheromone. Science.

[78] Siefkes MJ, Scott AP, Zielinski B, Yun S-S, Li W (2003). Male sea lampreys, Petromyzon marinus L., excrete a sex pheromone from gill epithelia. Biol Reprod.

[79] Johnson NS, Siefkes MJ, Wagner CM, Dawson H, Wang H, Steeves T (2013). A synthesized mating pheromone component increases adult sea lamprey (Petromyzon marinus) trap capture in management scenarios. Can J Fish Aquat Sci.

[80] Yun S-S, Scott AP, Li W (2003). Pheromones of the male sea lamprey, Petromyzon marinus L.: structural studies on a new compound, 3-keto allocholic acid, and 3-keto petromyzonol sulfate. Steroids.

[81] Li W (2005). Potential multiple functions of a male sea lamprey pheromone. Chem Senses.

[82] Johnson NS, Yun S-S, Li W (2014). Investigations of novel unsaturated bile salts of male sea lamprey as potential chemical cues. J Chem Ecol.

[83] Yeh C-Y, Chung-Davidson Y-W, Wang H, Li K, Li W (2012). Intestinal synthesis and secretion of bile salts as an adaptation to developmental biliary atresia in the sea lamprey. Proc Natl Acad Sci U S A.

[84] Cai S-Y, Lionarons DA, Hagey L, Soroka CJ, Mennone A, Boyer JL (2013). Adult sea lamprey tolerates biliary atresia by altering bile salt composition and renal excretion. Hepatology.

[85] Brant CO, Chung-Davidson Y-W, Li K, Scott AM, Li W (2013). Biosynthesis and release of pheromonal bile salts in mature male sea lamprey. BMC Biochem.

[86] Vrieze LA, Bergstedt RA, Sorensen PW (2011). Olfactory-mediated stream-finding behavior of migratory adult sea lamprey (Petromyzon marinus). Can J Fish Aquat Sci.

[87] Wagner CM, Twohey MB, Fine JM (2009). Conspecific cueing in the sea lamprey: do reproductive migrations consistently follow the most intense larval odour?. Anim Behav.

[88] Brant CO, Li K, Johnson NS, Li W (2015). A pheromone outweighs temperature in influencing migration of sea lamprey. R Soc Open Sci.

[89] Binder TR, McDonald DG (2007). The role of temperature in controlling diel activity in upstream migrant sea lampreys (Petromyzon marinus). Can J Fish Aquat Sci.

[90] Binder TR, McLaughlin RL, McDonald DG (2010). Relative importance of water temperature, water level, and lunar cycle to migratory activity in spawning-phase sea lampreys in Lake Ontario. T Am Fish Soc.

[91] Fine JM, Vrieze LA, Sorensen PW (2004). Evidence that petromyzontid lampreys employ a common migratory pheromone that is partially comprised of bile acids. J Chem Ecol.

[92] Yun S-S, Scott AP, Bayer JM, Seelye JG, Close DA, Li W (2003). HPLC and ELISA analyses of larval bile acids from Pacific and western brook lampreys. Steroids.

[93] Yun S-S, Wildbill AJ, Siefkes MJ, Moser ML, Dittman AH, Corbett SC (2011). Identification of putative migratory pheromones from Pacific lamprey (Lampetra tridentata). Can J Fish Aquat Sci.

[94] Robinson TC, Sorensen PW, Bayer JM, Seelye JG (2009). Olfactory sensitivity of Pacific lampreys to lamprey bile acids. T Am Fish Soc.

[95] Stewart M, Baker CF, Cooney T (2011). A rapid, sensitive, and selective method for quantitation of lamprey migratory pheromones in river water. J Chem Ecol.

[96] Buchinger TJ, Wang H, Li W, Johnson NS (2013). Evidence for a receiver bias underlying female preference for a male mating pheromone in sea lamprey. Proc R Soc B.

[97] Sorensen PW, Scott AP (1994). The evolution of hormonal sex pheromones in teleost fish: poor correlation between the pattern of steroid release by goldfish and olfactory sensitivity suggests that these cues evolved as a result of chemical spying rather than signal specialization. Acta Physiol Scand.

[98] Pfeiffer W, Riegelbauer G, Meier G, Scheibler B (1985). Effect of hypoxanthine-3 (N)-oxide and hypoxanthine-1 (N)-oxide on central nervous excitation of the black tetraGymnocorymbus ternetzi (Characidae, Ostariophysi, Pisces) indicated by dorsal light response. J Chem Ecol.

[99] Brown GE, Adrian JC, Smyth E, Leet H, Brennan S (2000). Ostariophysan alarm pheromones: laboratory and field tests of the functional significance of nitrogen oxides. J Chem Ecol.

[100] Mathuru AS, Kibat C, Cheong WF, Shui G, Wenk MR, Friedrich RW (2012). Chondroitin fragments are odorants that trigger fear behavior in fish. Curr Biol.

[101] Ferrero DM, Lemon JK, Fluegge D, Pashkovski SL, Korzan WJ, Datta SR (2011). Detection and avoidance of a carnivore odor by prey. Proc Natl Acad Sci U S A.

[102] Di Rocco RT, Belanger CF, Imre I, Brown GE, Johnson NS (2014). Daytime avoidance of chemosensory alarm cues by adult sea lamprey (Petromyzon marinus). Can J Fish Aquat Sci.

[103] Wisenden BD. Chemical cues that indicate risk of predation. In: Sorensen PW, Wisenden BD, editors. Fish Pheromones and Related Cues. John Wiley & Sons, Inc., Ames; 2015. p131-148.

[104] Perrault K, Imre I, Brown GE (2014). Behavioural response of larval sea lamprey (Petromyzon marinus) in a laboratory environment to potential damage-released chemical alarm cues. Can J Zool.

[105] Witzgall P, Kirsch P, Cork A (2010). Sex pheromones and their impact on pest management. J Chem Ecol.

[106] Sorensen PW. Applications of pheromones in invasive fish control and fishery conservation. In: Sorensen PW, Wisenden BD, editors. Fish Pheromones and Related Cues. John Wiley & Sons, Inc., Ames; 2015. p255-268.

[107] Johnson NS, Tix JA, Hlina BL, Wagner CM, Siefkes MJ, Wang H (2015). A sea lamprey (Petromyzon marinus) sex pheromone mixture increases trap catch relative to a single synthesized component in specific environments. J Chem Ecol.

[108] Johnson NS, Siefkes MJ, Li W (2005). Capture of ovulating female sea lampreys in traps baited with spermiating male sea lampreys. N Am J Fish Manage.

[109] Wagner CM, Jones ML, Twohey MB, Sorensen PW (2005). A field test verifies that pheromones can be useful for sea lamprey (Petromyzon marinus) control in the Great Lakes. Can J Fish Aquat Sci.

[110] Luehring MA, Wagner CM, Li W (2011). The efficacy of two synthesized sea lamprey sex pheromone components as a trap lure when placed in direct competition with natural male odors. Biol Invasions.

[111] Johnson NS, Siefkes MJ, Wagner CM, Bravener G, Steeves T, Twohey M, Li W. Factors influencing capture of invasive sea lamprey in traps baited with synthesized sex pheromone components. J Chem Ecol. In press. 10.1007/s10886-015-0626-2 26399432

[112] Hume JB, Meckley TD, Johnson NS, Luhring TM, Siefkes MJ, Wagner CM. Application of a putative alarm cue hastens the arrival of invasive sea lamprey (Petromyzon marinus) at a trapping location. Can J Fish Aquat. 2015; published online 28July2015.

[113] Xi X, Johnson NS, Brant CO, Yun S-S, Chambers KL, Jones AD (2011). Quantification of a male sea lamprey pheromone in tributaries of Laurentian Great Lakes by liquid chromatography–tandem mass spectrometry. Environ Sci Technol.

[114] Wang H, Johnson NS, Bernardy J, Hubert T, Li W (2013). Monitoring sea lamprey pheromones and their degradation using rapid stream‐side extraction coupled with UPLC‐MS/MS. J Sep Sci.

[115] Johnson NS, Thompson HT, Holbrook C, Tix JA (2014). Blocking and guiding adult sea lamprey with pulsed direct current from vertical electrodes. Fish Res.

[116] Smith JJ, Kuraku S, Holt C, Sauka-Spengler T, Jiang N, Campbell MS (2013). Sequencing of the sea lamprey (Petromyzon marinus) genome provides insights into vertebrate evolution. Nature Genet.

[117] Walaszczyk EJ, Johnson NS, Steibel JP, Li W (2014). Effects of sex pheromones and sexual maturation on locomotor activity in female sea lamprey (Petromyzon marinus). J Biol Rhythm.

[118] Sorensen PW, Hoye TR (2007). A critical review of the discovery and application of a migratory pheromone in an invasive fish, the sea lamprey Petromyzon marinus L. J Fish Biol.

